# What Is New in Altitude- and Cold-Related Illnesses of Travel: Appraisal and Summary of the Updated Guidelines from the Wilderness Medical Society

**DOI:** 10.3390/ijerph22020284

**Published:** 2025-02-14

**Authors:** Arghavan Omidi, Gregory D. Hawley, Dylan Kain, Farah Jazuli, Milca Meconnen, Mark Polemidiotis, Nam Phuong Do, Olamide Egbewumi, Andrea K. Boggild

**Affiliations:** 1 Faculty of Dentistry, University of Toronto, Toronto, ON M5G 1G6, Canada; 2 Division of Infectious Diseases, Department of Medicine, University of Toronto, Toronto, ON M5S 3H2, Canada; 3 Pulmonary, Allergy & Critical Care Medicine, School of Medicine, Oregon Health & Sciences University, Portland, OR 97239-3098, USA; 4 Department of Medicine, Division of Emergency Medicine, McMaster University, Hamilton, ON L8L 2X2, Canada; 5 Department of Biology, McMaster University, Hamilton, ON L8S 4K1, Canada; 6 Institute of Medical Science, University of Toronto, Toronto, ON M5S 3H2, Canada; 7 School of Medicine, Royal College of Surgeons in Ireland, D02 YN77 Dublin, Ireland; 8 Department of Medicine, University of Toronto, Toronto, ON M5S 3H2, Canada; 9 Tropical Disease Unit, Toronto General Hospital, Toronto, ON M5G 2C4, Canada

**Keywords:** acute mountain sickness, avalanche, frostbite, high-altitude cerebral edema, high-altitude pulmonary edema, Wilderness Medicine Society

## Abstract

Wilderness medicine is a rapidly evolving field and has benefited from expanded research efforts. Moreover, with an escalating occurrence of severe and cataclysmic global climatologic events, human illness arising from interaction with wilderness and recreational environments warrants increasing consideration. Within the last decade, the Wilderness Medical Society (WMS) has aggregated research findings and created guidelines on prevention measures and therapeutic options for acute altitude illness, frostbite injuries, and avalanche and non-avalanche snow burials. As new research emerges, some guidelines have been updated to reflect the most current and sound scientific conclusions. In this review, we have synthesized the evidence-based guidelines and have reviewed the quality of the guidelines according to the Appraisal of Guidelines for Research and Evaluation (AGREE) II framework. Further research efforts can expand the scope of evidence-based practice in travel medicine and ideally standardize the implementation of recommendations within both pre-travel and post-travel medical practices.

## 1. Introduction

Global rates of travel have skyrocketed in recent years, and with this, travelers have also become increasingly adventurous, placing themselves into ever-more extreme altitudes, climates, and ecologies. Additionally, extreme climatologic events coupled with a warming planet destabilize glaciers and mountaineering environments, which place individuals at elevated risk of phenomena such as avalanche and snow burials. The global adventure tourism market was valued at USD 586.3 billion in 2018 but is projected to reach USD 1.63 trillion in 2026, showing a growth of 13.3% [1]. With this rising trend, travelers are at an increased risk of environmental illness and exposures; travel specialists must therefore develop expertise in environmental illness. Such practice fluency involves both being up to date on the evidence-based treatment of environmental illness as well as understanding how to mitigate risk, such that appropriate counselling of patients during pre-travel medical visits may occur. The Wilderness Medicine Society (WMS) has recently published updates on four practice guidelines, summarizing the evidence for the prevention and treatment of acute altitude sickness [2], frostbite [3], heat illness [4], and avalanche and non-avalanche snow burial [5]. Here, we provide a summary of the practice guidelines for acute altitude illness, frostbite, and snow burials and their corresponding grading as well as an appraisal of evidence provided for each topical guidance. This review is intended to update and guide travel medicine specialists caring for patients at risk of environmental exposures as well as raise awareness amongst the general medical and public health communities around environmental illnesses that may be encountered with escalating and severe climatologic events globally.

### 1.1. Summary and Critical Appraisal

The practice guidelines and focus of research advancement for all systematic reviews are summarized in this paper with an emphasis on the updated literature. Three systematic reviews with updated guidelines [2,3,5] were assessed for the quality of evidence giving rise to each practice guideline. The Appraisal of Guidelines for Research and Evaluation (AGREE) II instrument was used by two authors to assess methodological rigor and transparency in development of the guidelines collectively [6,7]. The AGREE II instrument comprises a six-domain, 23-item checklist that is scored on a numeric scale from 1 to 7 across items within each domain [6,7]. A full user’s manual with step-by-step instructions on how to apply and score the instrument is available at www.agreetrust.org (accessed on 26 January 2025). Composite and aggregate scaled scores across the six AGREE II domains are provided in Supplementary Table S1. A summary of preventive and therapeutic interventions recommended in the guidelines is provided in Table 1 and Table 2. The guidelines the authors adhered to were the American College of Chest Physicians (ACCP) updated classification scheme for recommendations in clinical guidelines, balancing risks and benefits with burden of evidence, which enabled assignment of both a grading of quality of evidence and a strength of recommendation.

### 1.2. Assessment of Methodological Rigor and Transparency According to AGREE II [6,7] Criteria

Aggregate and scaled scores for the first domain of AGREE II, “Scope and Practice”, were 5.7/7 and 78%, respectively (Supplementary Table S1). Although sufficient methodological detail was provided to infer the target population and assessed interventions, notably absent from the guidelines were clear “PICO” (population–intervention–comparator–outcome) questions. As such, it is unclear if the scope of the guidelines pertains to both adults and children; however, the altitude guidelines explicitly state that pediatric-related data were captured by the search strategy. The objectives of the guidelines are clearly described.

Aggregate and scaled scores for the second domain of AGREE II, “Stakeholder Involvement”, were lowest in this domain—4.7/7 and 61%, respectively—due to a lack of expressed involvement in patients and/or the public in the development of the guidelines (Supplementary Table S1). Based on authorship representation, however, clear involvement of international experts and those with specialized expertise in search, rescue, and recovery were clearly engaged and involved in the guideline development process. On the other hand, all relevant stakeholder medical specialties were not represented, with a notable absence of representation from pharmacology, allied and rehabilitation services, and pediatrics and pediatric intensivists. Clarity around the exact intended audience would enhance understanding on this domain in particular.

Aggregate and scaled scores for the third domain of AGREE II, “Rigor of Development”, were 5.9/7 and 82%, respectively (Supplementary Table S1). Evidence was synthesized in a systematic, transparent manner for each of the guidelines, and the strengths and limitations of the evidence base are described. The guidelines would benefit from further exposition around language and date restrictions as well as the precise search strategy employed. A clear and rigorous process for formulating recommendations according to quality and strength was undertaken in each case. Both the risks and benefits of each intervention are considered, and the guidelines have been peer-reviewed. Although there is no explicit text describing the process for updating the guidelines, one can infer that this is planned due to the existence of prior iterations.

Aggregate and scaled scores for the fourth domain of AGREE II, “Clarity of Presentation”, were highest at 7/7 and 100%, respectively (Supplementary Table S1). The recommendations are clear, unambiguous, and easily identified throughout each of the guidelines. Different management options are presented clearly throughout each guideline.

Aggregate and scaled scores for the fifth domain of AGREE II, “Applicability”, were also lower than some other domains due to a lack of explicit consideration of barriers to implementation and strategies to encourage adoption across intended stakeholder groups (Supplementary Table S1). The aggregate score was 6/7 across the items, with a scaled score of 83%. Biological and physiological benchmarks are clear and provide quantitative metrics by which auditing of practice could occur in each case (e.g., oxygen saturation targets, tissue viability correlates, etc), although a process by which to undertake such monitoring is not described. There are excellent clinical resources and algorithms provided in both the altitude and avalanche guidelines, and usable summary tables for guidance provided in all.

Aggregate and scaled scores for the sixth domain of AGREE II, “Editorial Independence”, were again highest in this domain due to the clear lack of influence on the part of the funders and those with competing interests (Supplementary Table S1). Aggregate scores of 7/7 were achieved across the items, with a scaled score of 100%.

The overall quality of the guidelines, collectively and in aggregate, was rated as 6/7, with a scaled score of 83%, and the authors applying the AGREE II instrument unanimously agreed that the guidelines should be recommended for use (Supplementary Table S1).

### 1.3. Definitions and Epidemiology of Acute Altitude Illness Highlighted in the Guidelines

Acute altitude illness includes several different syndromes, including acute mountain sickness (AMS), high-altitude cerebral edema (HACE), and high-altitude pulmonary edema (HAPE). Travel to elevations above 2500 m is associated with increased risk of developing acute altitude illness [8]. Such elevations are encountered in many popular travel destinations, including Machu Picchu and the Bolivian Salt Flats, as well as recreational travel specifically for the purpose of visiting high-altitude landmarks such as Everest base camp or climbing Mount Kilimanjaro. Travel health specialists are often asked about the prevention and treatment of acute altitude illnesses and can therefore serve as a possible point of intervention to reduce altitude illness in travelers. Moreover, the trend of increasingly extreme adventure expeditions on the itinerary of travelers necessitates familiarity with potential environmental illnesses on the part of the general medical and public health communities.

Travel to high altitudes leads to a drop in barometric pressure, causing a decrease in the partial pressure of oxygen and, thus, oxygen delivery at the mitochondrial level. There are a series of physiological responses designed to counteract this, but they are capped to limited altitudes, even in healthy individuals. There is a wide tolerance throughout the population for the pressure that will cause failure of these mechanisms, with some otherwise healthy people having symptoms of acute altitude illness around 2000 m. Of note is the fact that many popular mountain ski resorts are located above this altitude.

AMS consists of nonspecific symptoms that generally occur above 2500 m in an unacclimated individual, usually within 4–12 h and up to 72 h of arrival at that altitude. As per the Lake Louise guidelines, the criteria for AMS diagnosis include altitude-related headache as well as one of the following: gastrointestinal symptoms, fatigue/weakness, or dizziness/lightheadedness [8]. AMS can progress to the more severe HACE, for which the leading signs are truncal ataxia and clouded consciousness [8]. Without appropriate treatment, HACE can lead to death in as little as 24 h.

HAPE usually occurs after 1–5 days of arrival at altitudes over 2500–3000 m and is extremely rare below these levels or after 1 week of acclimatization to these altitudes [8]. It is often preceded by AMS. The diagnosis of HAPE requires two of the following symptoms: chest tightness or pain, dyspnea at rest, cough, and decreased exercise tolerance, as well as two of the following exam findings: crackles/wheezes, cyanosis, tachypnea, and/or tachycardia [9].

### 1.4. Strategies for Prevention of Altitude Illness Outlined in the Guidelines

The 2024 update of WMS guidelines are derived from recent evidence based on the newer search terms of budesonide, acetaminophen, continuous positive airway pressure (CPAP), and hypoxic tents. Previously recommended prevention strategies against AMS and HACE remain intact, which include gradual ascent (strong recommendation, moderate-quality evidence); the use of 125 mg of acetazolamide every 12 h starting the night before ascent (strong recommendation, high-quality evidence); the use of 2 mg of dexamethasone every 6 h (strong recommendation, high-quality evidence); the use of ibuprofen in the case of inability to use acetazolamide and dexamethasone (weak recommendation, moderate-quality evidence); staged ascent and preacclimatization (strong recommendation, low-quality evidence); as well as other options such as consumption of coca products. The herbal medicine Gingko biloba is not recommended for AMS prevention, as trials have demonstrated inconsistent results due to variation in herb sources, and as such, caution is advised without knowledge of its source and composition (strong recommendation, low-quality evidence). New evidence suggests the beneficial effects of budesonide inhalation for AMS prevention [10,11], but the results were limited by methodological issues and were not replicable [12,13], leading to the recommendation that inhaled budesonide should not be used for altitude-related prophylaxis (strong recommendation, high-quality evidence). Similarly, methodological issues, namely, improper control measures, have arisen in new evidence pertaining to acetaminophen use as a prevention measure against AMS [14]. These issues led to new recommendations discouraging acetaminophen use in this context (strong recommendation, low-quality evidence). There are only two placebo-controlled studies on the use of hypoxic tents, and neither has shown a statistically significant reduction in the incidence of acute mountain sickness, preacclimatization, or increasing performance to reach the summit [2,15]. As a result, the use of hypoxic tents is not recommended in this context (strong recommendation, moderate-quality evidence). Although in the 2019 review of the guidelines the recommendation to implement prevention strategies was linked to risk status, with risk factors including history of altitude illness and altitude of ascent, the 2024 guidelines propose a less stratified approach in risk identification and thus a broader application of prevention strategies [2].

### 1.5. Interventions for Treatment of Altitude Illness Evaluated in the Guidelines

Recommendations for therapeutic strategies to alleviate AMS and HACE symptoms still include altitude descension (strong recommendation, high-quality evidence), use of supplemental oxygen targeting blood oxygen (SpO2) > 90% (strong recommendation, high-quality evidence), use of portable hyperbaric chambers for severe cases or inability to descend (strong recommendation, moderate-quality evidence), use of dexamethasone 8 mg followed by 4 mg every 6 h (strong recommendation, low-quality evidence), and acetazolamide 250 mg every 12 h (strong recommendation, low-quality evidence). New evidence suggests that ibuprofen and acetaminophen can relieve headaches at high altitude, but their therapeutic effects are limited to just that and are therefore not suggested for use to treat AMS and HACE (strong recommendation, low-quality evidence) [16]. Continuous positive airway pressure (CPAP), which increases alveolar volume and thus improves pulmonary gas exchange, has demonstrated feasibility as a potential therapeutic measure for AMS [17,18], but due to limited systematic research, it cannot be recommended as treatment. In summary, the 2024 guidelines for AMS and HACE treatment prioritize the avoidance of further ascent along with close monitoring and symptomatic management of mild-to-moderate illness. However, in persistent symptoms or moderate–severe cases, altitude descent is prioritized if feasible, along with adjunctive acetazolamide, dexamethasone, and supplemental oxygen if available.

HAPE may follow AMS in certain individuals and presents with respiratory symptoms. Updated WMS guidelines for its prevention remain quite similar regarding strategies such as gradual ascent (strong recommendation, moderate-quality evidence), use of nifedipine 30 mg extended release every 12 h (strong recommendation, moderate-quality evidence), use of tadalafil as an alternative to nifedipine (strong recommendation, low-quality evidence), use of dexamethasone if nifedipine and tadalafil cannot be administered (strong recommendation, low-quality evidence), use of acetazolamide by children for prevention of AMS (strong recommendation, low-quality evidence), reentry HAPE (strong recommendation, low quality evidence), and preacclimatization through staged ascent when feasible (strong recommendation, low-quality evidence). However, the WMS no longer recommends salmeterol as a supplement to nifidepine, given that the dosage deemed effective by single randomized, placebo-controlled study [19] in reducing incidence of HAPE also has many unwanted side effects associated with it. In fact, more current research still calls for randomized control trials investigating the effectiveness of salmeterol as a pulmonary vasodilator [20].

As for HAPE treatment, recommendations regarding descent (strong recommendation, high-quality evidence) and the use of supplemental oxygen (strong recommendation, high-quality evidence) or portable hyperbaric chambers when descent is not feasible or delayed or supplemental oxygen is unavailable (strong recommendation, low-quality evidence) remain the highest rated. Phosphodiesterase inhibitors such as tadalifil or sidenafil are recommended as therapeutic agents but only in the absence of other interventions (weak recommendation, low-quality evidence). Additionally, similar to the 2014 review on altitude illness [21], the WMS cannot make recommendations on the use of beta-agonists and dexamethasone due to insufficient evidence and advises against the use of diuretics due to the risk of intravascular volume depletion in HAPE patients (strong recommendation, low-quality evidence). CPAP may be considered as an alternate or adjunctive avenue of therapy but is not highly recommended due to a lack of systematic evidence and limitations on its overall utility and feasibility (weak recommendation, low-quality evidence). Nifedipine is recommended only as an alternative solution in the absence of supplemental oxygen, as new evidence from a prospective cross-sectional study about nifedipine use as an adjunct to descent and supplemental oxygen showed no additional benefits (strong recommendation, low-quality evidence) [22]. Updated guidelines recommend against the use of acetazolamide due to a lack of evidence from systematic studies as well as a suspected detrimental diuretic effect on HAPE patients (strong recommendation, moderate-quality evidence). The 2024 update to the guidelines for treating HAPE prioritizes oxygenation of affected individuals within the treatment sequence followed by descent and the aforementioned pharmacological interventions in the event of stagnant and/or progressive symptoms [2].

The WMS provides no updates on guidelines for therapeutic approaches to take regarding patients with concurrent HAPE and HACE. Dexamethasone at a dosage of 2 mg every 6 h or 4 mg every 12 h should be added to the treatment regimen [2], with caution being taken with HAPE patients with neurologic dysfunction, which may often be misdiagnosed as patients suffering from HACE.

The 2024 update of WMS guidelines on acute altitude illness recognizes the impact of the COVID-19 pandemic on travel trends. In addition, those who may experience cardiopulmonary complications as a result of COVID-19 may be at greater risk of altitude illness and emergencies. As such, careful cardiac and respiratory evaluation in those with persistent COVID-19 symptoms, events of COVID-19 related myocarditis, or thromboembolic disease is warranted [2].

Through expert consensus, the WMS has provided thorough updated recommendations on AMS, HACE, and HAPE prevention and management strategies such as, but not limited to, pharmaceutical interventions.

### 1.6. Definitions and Epidemiology of Frostbite Highlighted in the Guidelines

Adventure travel to cold climates coupled with increasingly extreme and unpredictable weather events places travelers at risk of frostbite. This is common in those who travel to high altitudes, where the temperature is often well below freezing; however, frostbite also occurs at lower altitudes, for example, when travelers visit cold destinations for winter activities, such as skiing, or when they visit geographies with large and unexpected temperature swings, such as deserts. Frostbite also occurs in contexts outside of travel, specifically in individuals living in cold climates. Individuals without stable housing are at highest risk in this setting.

Frostbite is a freezing injury that represents a spectrum of four phases: prefreeze, freeze–thaw, vascular stasis, and late ischemia. In prefreeze, there is no actual ice crystal formation, but instead, there is tissue cooling with associated vasoconstriction and ischemia. Hyperesthesia or paresthesia can occur due to neuronal cooling and ischemia. In the freeze–thaw phase, ice crystals form in the tissue, leading to cell death. During the thawing phase, a reperfusion injury and inflammatory response can occur. During the vascular stasis injury phase, there is plasma leakage from vessels, as well as vasospasm and stasis coagulation. In late ischemia, there is progressive tissue ischemia and infarction [3].

Frostnip is a superficial non-freezing injury that often precedes frostbite but is a distinct entity. Similar to thermal burn injuries, frostbite can be classified based on the depth of the injury [21]:

- First-degree frostbite leads to erythema and numbness. There is no gross tissue infarction, and damage is not generally permanent.
- Second-degree frostbite leads to blisters forming that can be filled with milky fluid. There can be lasting numbness and cold sensitivity.
- Third-degree frostbite results from freezing of tissue below the layers of skin. There can be blue–grey discolouration of the skin, and weeks after the injury, pain can persist and an eschar can form. Patients can have long-term ulceration and damage to the growth plates.
- Fourth-degree frostbite results from freezing in deeper structures, such as the muscles, tendon, or bone. The skin becomes colorless and hard, and there is painless rewarming. Later, the skin becomes black and mummified before permanent damage leads to autoamputation over several months.

For field classification, after rewarming but before imaging, an alternate two-tiered system is favored. Frostbite can be classified as superficial, with no or minimal anticipated tissue loss (corresponding to first- and second-degree injury), or deep, with anticipated tissue loss (corresponding to third- and fourth-degree injury).

The 2024 updated WMS guidelines outline one additional in-hospital method of classification of post-thaw frostbite injury: the Cauchy classification [23] method. The Cauchy classification method uses a graded system, ranging from 0–5, to determine the extent of anatomical frostbite injury, where higher grades correspond to more proximal injuries. The overall severity of injury is then graded on a scale from 1–4. This method assists caregivers in predicting amputation risk and informing evacuation decisions. The Hennepin score [24], which measures frostbite injury severity based on total body surface area (similar to measuring thermal burns) was included in the 2019 guidelines but is no longer used as a method of classification in the updated 2024 guidelines.

### 1.7. Strategies for the Prevention of Frostbite Outlined in the Guidelines

The WMS emphasizes the importance of prevention measures against frostbite injuries, as treatment interventions are less likely to be fully restorative. Three main categories of prevention strategies are outlined in the guidelines, namely, maintaining peripheral perfusion, exercise, and protection from the cold. Peripheral perfusion guidelines aim to prevent factors that may compromise blood flow, such as drugs, alcohol, and constrictive clothing, and to maintain peripheral perfusion through adequate nutrition and oxygenation (strong recommendation, low-quality evidence). Exercise is recommended as a method of promoting peripheral perfusion, as evidence suggests an increase in extremity vasodilation following physical activity (strong recommendation, moderate-quality evidence) [25,26]. As for protection from the cold, the guidelines recommend appropriate outerwear in cold temperatures, the use of electrical and chemical warmers, and cautionary measures such as minimizing the duration of exposure to cold (strong recommendation, low-quality evidence). The 2024 WMS guidelines now recommend avoidance of environmental conditions that predispose to frostbite as less than 0 °C (some risk at 0 to –15 °C, increasing risk below −15 °C), whereas the 2019 guidelines recommended avoidance of conditions less than −15 °C.

### 1.8. Interventions for Treatment of Frostbite Evaluated in the Guidelines

Recommendations for field treatment and secondary prevention of frostbite injuries mainly revolve around avoiding refreezing of tissue, as that triggers further thrombosis and cellular injury (strong recommendation, moderate-quality evidence) [27,28,29]. Treatment guidelines are presented for two scenarios: (1) frozen tissue is not actively thawed due to potential of refreezing, or (2) frozen tissue is thawed with no further risk of refreezing. In the first scenario, where active thawing cannot occur, immediate evacuation from the cold should be prioritized, and the patient should seek medical care. Expert opinion from the WMS panel suggests that the affected area should be immobilized, or if mobility is unavoidable, that the frozen area should be well padded, splinted, and kept as immobile as possible to prevent further trauma (weak recommendation, very-low-quality evidence). No evidence supports dressings to the frozen part, but if one is considered, a dry clean gauze that does not restrict mobility should be applied (weak recommendation, very-low-quality evidence). Alternatively, if active thawing is feasible, rapid rewarming with continuous warm water bath immersion, at a temperature of 37 to 39 °C, should be performed for approximately 30 min or until the affected tissue becomes erythematous and pliable (strong recommendation, moderate-quality evidence). If active field rewarming cannot be performed, passive or spontaneous thawing is recommended (strong recommendation, low-quality evidence). Secondary treatment strategies include the application of antiseptics to rewarming water (weak recommendation, very-low-quality evidence), dressings (strong recommendation, low-quality evidence), and topical aloe vera (weak recommendation, very-low-quality evidence). Other priorities include administration of pain-control agents (strong recommendation, low-quality evidence), supplemental oxygen in hypoxic patients (oxygen saturation < 88%) or at altitude > 4000 m (weak recommendation, low-quality evidence), limited debridement of blisters (weak recommendation, low-quality evidence), elevation of extremities (strong recommendation, low-quality evidence), and immobilization of the affected limb (weak recommendation, low-quality evidence), as recommended based on expert opinion and/or observational and case studies [30,31,32,33]. Frostbite injuries may be accompanied by mild hypothermia, vascular stasis, and hypovolemia, all of which can be treated concurrently with thawing of frostbite injuries (strong recommendation, low-quality evidence). Moderate and severe hypothermia, however, should be treated before management of frostbite injuries. Evidence from animal trials [31,34,35,36,37,38] supports the administration of antithrombotic agents, including low-molecular-weight dextran (LMWD) and ibuprofen. LMWD should be administered when systemic therapies, such as thrombolytics or iloprost, are not available or not feasible (weak recommendation, low-quality evidence). The low risk of anaphylaxis and minimal risk of bleeding, where benefits appear to outweigh risks, should not deter the administration of LMWD. Ibuprofen should be administered routinely in the field at a dose of 12 mg/kg/day divided twice daily to a maximum of 2400 mg/day divided four times daily (weak recommendation, low-quality evidence). There have been no changes to field management strategies in the 2024 update.

Initial hospital management of frostbite injuries is summarized in Table 1. Since the 2019 review of frostbite injury management, updates have been made to the guidelines regarding thrombolytics, imaging, and heparin as well as the newly added recommendation for the usage of iloprost, a prostacyclin analogue. As of 2022 inclusive, the recommendation for immediate intravenous (IV) or intra-arterial (IA) thrombolytic therapy for deep frostbite injuries has been further validated through evidence from one randomized controlled prospective trial (tPA plus iloprost, *n* = 16) [39], one retrospective observational study (*n* = 125) [40], three retrospective cohort studies (*n* = 59) [41,42,43], eleven retrospective case series (*n* = ~200) [44,45,46,47,48,49,50,51,52,53,54], and three case reports [55,56,57]. The WMS guidelines recommend the administration of IV or IA tPA for the treatment of severe frostbite (Cauchy Grade 3 or 4) within 24 h of injury, especially in cases where iloprost is unavailable (strong recommendation, low-quality evidence). New in the 2024 guidelines is that tPA started early at remote community hospitals prior to transfer to tertiary care centers with appropriate intensive care monitoring capabilities may safely decrease rates of amputation [58] (strong recommendation, low-quality evidence). The 2024 guidelines have also clarified dosing regimens for both routes of administration. IV tPA is typically dosed at 0.15 mg/kg bolus followed by 0.15 mg/kg/h for six hours with concurrent heparin for 3–5 days. Although IA dosing varies, a typical regimen is a 2–4 mg bolus followed by 1 mg/h for 24–48 h, with concurrent heparin or LMWH. The WMS guidelines for imaging as a means of assessing possible amputation dimensions have been updated to include single photon emission computed tomography (CT)/CT as a possible technique, as per evidence provided by Kraft and colleagues in a study assessing the ability of this technique to enable early and precise anatomic localization of nonviable tissue in seven frostbite patients [59,60]. New to the 2024 guidelines is a recent study that utilized multiphase [Tc99m]-MDP bone scintigraphy with SPECT/CT in the setting of severe frostbite injury [61]. The updated guidelines continue to recommend the use of appropriate imaging to evaluate tissue viability and guide decisions around amputation, where available (weak recommendation, low-quality evidence). Research into the use of iloprost, a potent vasodilator, by the WMS panel was inspired by the first account of intravenous iloprost therapy for frostbite patients, which resulted in recovery without amputation in 100% of the patients [62]. Randomized trials and case series demonstrated that iloprost showed favorable effects if administered within 72 h of injury, with no serious side effects reported afterwards [39,46,63,64]. In the updated 2024 guidelines, iloprost is recommended for deep frostbite extending to the distal interphalangeal joint or proximal (Cauchy Grades 2–4) within 72 h of injury (strong recommendation, low-quality evidence) [64,65,66]. In the 2019 guidelines, a starting administration rate of 0.5 ng/kg/min with a gradual increase to a maximum rate of 2.0 ng/kg/min for 6 h daily is suggested; however, no dosing regimen is listed in the 2024 guidelines. Updates on the duration of iloprost therapy suggest a reasonable starting point of 5–8 days, which can be shortened or extended based on imaging findings of tissue reperfusion. Both sets of guidelines comment on the lack of evidence surrounding the use of heparin or LMWH as a monotherapy in frostbite management; as such, heparin monotherapy is not recommended (weak recommendation, low-quality evidence). The 2024 guidelines now support this recommendation due to low-quality evidence rather than insufficient data, as listed in the 2019 guidelines. Although heparin is used concurrent with tPA protocols, as described above, there are insufficient data to recommend its use with iloprost.

Post-thaw medical interventions that are recommended include hydrotherapy (strong recommendation, low-quality evidence), hospitalization for deep frostbite or monitoring of complications and high-risk individuals (weak recommendation, low-quality evidence), fasciotomy in cases of compartment syndrome (strong recommendation, low-quality evidence), and surgical interventions and amputation if deemed necessary (strong recommendation, low-quality evidence). Hyperbaric oxygen therapy cannot be recommended due to insufficient evidence; however, retrospective data and a prospective case-control study demonstrate improvement in wounds following hyperbaric oxygen [3,39,67]. New to the 2024 guidelines is a recommendation to not perform sympathectomy (weak recommendation, low-quality evidence). Previously, in 2019, no recommendation was made due to insufficient evidence.

Frostbite injuries have the potential to cause long-term, irreversible detriments. Although there are many effective frostbite management strategies that can reduce injury and risk of morbidity, the updated WMS guidelines identify several areas for future research. These include better elucidation of frostbite pathophysiology, medications for prevention of frostbite, peri-thawing procedures to minimize injury, and post-thawing therapies to improve long-term outcomes.

### 1.9. Definitions and Epidemiology of Avalanche and Non-Avalanche Snow Burial Highlighted in the Guidelines

Globally, mountainous regions at risk for avalanches are numerous and attract many visitors. In the context of a warming planet and extreme weather events, glacier regions and hard-packed snow are increasingly destabilized, placing adventurers at risk of avalanche and burials. Snowmobilers, skiers, snowboarders, mountaineers, and snowshoers are among the groups with the highest number of documented avalanche fatalities in North America and Europe [5]. For others, such as mountain guides, rescuers, or ski patrols, the risk is occupational. Approximately 75% of avalanche fatalities are attributed to asphyxiation, which can occur through snow inhalation, ice mask formation, or oxygen deprivation. Close to 25% can be attributed to major injuries of the head, spine, and extremities. Other cases can be explained by mild, moderate, or severe hypothermia, as graded by WMS practice guidelines. As avalanche-related injuries are of high severity in their nature, the WMS has presented guidelines pertaining to prevention, rescue, and resuscitation. Pre-travel practitioners should have a basic understanding of these methods to allow for better counselling of patients prior to travel.

### 1.10. Strategies for Prevention of Avalanche and Snow Burial Outlined in the Guidelines

Prevention guidelines fall within the categories of avalanche avoidance, burial avoidance, trauma minimization, and asphyxia avoidance. Avalanche avoidance can be facilitated by appropriate training and knowledge of terrains and avalanche routes (strong recommendation, low-quality evidence). To avoid burial as a result of an avalanche, expert consensus recommends that travelers practice slope cutting, a method of testing slope stability, and carry belays, which are a type of anchor. Additionally, based on case studies and theoretical considerations, it is recommended to maintain physical movements in order to counteract the flow of an avalanche in an attempt to avoid burial (strong recommendation, low-quality evidence) [68]. Avalanche airbags, which have shown an 11% reduction in mortality rate in retrospective studies of avalanche accident records from Europe and North America, are recommended as an essential item to carry and become well familiarized with prior to entering high-risk areas (strong recommendation, moderate-quality evidence) [69]. Use of helmets can theoretically reduce the risk of traumatic brain injuries secondary to avalanche events, although there is a paucity of evidence in the literature regarding this (strong recommendation, low-quality evidence). In the event that one is caught in an avalanche, it is recommended to create an air pocket manually through covering of the mouth to reduce the risk of asphyxiation (strong recommendation, low-quality evidence), or with the use of an avalanche airbag (strong recommendation, low-quality evidence). Alternatively, an artificial air pocket device can be used, which diverts exhaled carbon dioxide and can prevent ice-mask formation (strong recommendation, low-quality evidence). Although no studies have been conducted to compare mortality amongst users and nonusers, a randomized, crossover-controlled trial has demonstrated that usage of an artificial air pocket device can allow for sufficient oxygenation for up to 60 min [70].

### 1.11. Interventions for Treatment of Avalanche and Snow Burial Evaluated in the Guidelines

Rescue of avalanche victims is recommended to follow a sequence involving establishing a team and outlining a clear plan. The rescue team must first establish leadership and secure the scene prior to performing a surface search. Following a surface search, transceiver searches are recommended, given that victims also carry transceivers, as evidence shows that this results in a reduction in burial time from 102 min to 20 min, resulting in reduction in mortality rate from 68% to 54% (strong recommendation, moderate-quality evidence) [71]. Location-specific methods, such as probe searching (strong recommendation, low-quality evidence) and strategic shuffling, are recommended as the sequential next step. From there, rescuers can proceed by shoveling strategically, applying medical care, and evacuating the victims. Emergency services can be notified at a time deemed appropriate by the rescue team, such that it does not interfere with time-sensitive steps. Within professional rescue settings, it is recommended to employ a standardized command approach, termed an Incident Command System, to systematically delegate roles and apply adjunct search strategies such as usage of probe lines, RECCO radar systems, canine searches, and helicopter recoveries (strong recommendation, low-quality evidence). In the event of multiple burials, rescue teams should adhere to the recommendations of the International Commission of Mountain Emergency Medicine. Teams may use search-and-excavate protocols such as AvaLife, which involves search and excavation, followed by out-of-hospital medical treatment and then hypothermia staging and initial cardiopulmonary resuscitation (CPR) prior to transport of patients to a medical facility (weak recommendation, low-quality evidence).

Medical interventions to be applied upon discovery of an avalanche victim depend highly upon prognostic factors such as severity of injuries, duration of complete burial, core temperature, airway patency, and serum potassium levels. Situational guidelines have been made to optimize cardiopulmonary resuscitation (CPR) and extracorporeal life support (ECLS) efforts, as prolonged and unjustified efforts still lead to low survival rates [72,73,74]. The severity of injury (if present), duration of complete burial, airway patency, core temperature, initial serum potassium, and the Hypothermia Outcome Prediction after extracorporeal life support scores are just some of the prognostic factors to be considered for ECLS efforts. Resuscitation should be initiated after assessing life-threatening injuries that need primary attention. Next, core temperature and duration of burial should be investigated; if duration of burial is ≤60 min and core temperature is ≥30 °C, standard CPR should be initiated and performed for 30 min, after which attempts may be ceased due to low chances of survival (strong recommendation, moderate-quality evidence). If the duration of burial for a victim with a patent airway is >60 min and core temperature is <30 °C, cardiac arrest may be attributed to hypothermia, and therefore ECLS should be pursued along with CPR for as long as necessary (weak recommendation, moderate-quality evidence). Defibrillation is another recommended treatment for cardiac arrest avalanche victims, and clinical and experimental evidence suggests that the best results arise from defibrillation of victims with a higher core temperature (≥30 °C). However, victims with a core temperature <30 °C may still be defibrillated 1–3 times, and if unsuccessful, they may be administered standard CPR until core temperature raises (strong recommendation, moderate-quality evidence). In addition, unconscious victims may benefit from endotracheal intubation as a means of assisted ventilation, especially if hypercapnia is suspected (strong recommendation, moderate-quality evidence), in addition to mechanical chest compressions, specifically during difficult and long transports (strong recommendation, low-quality evidence). In victims with a much lower core temperature (20–28 °C), delayed or intermittent resuscitation with 5–10 min rest periods is recommended, as supported by evidence from case studies (weak recommendation, low-quality evidence) [75,76,77]. In conditions where the victim has sustained a traumatic cardiac arrest (weak recommendation, low-quality evidence) or is found after over 60 min of burial, pulseless, and apneic or in asystole with a core temperature ≥ 30 °C or with a core temperature <30 °C and an obstructed airway, resuscitation may be withheld or terminated (strong recommendation, moderate-quality evidence). In addition, a serum potassium concentration of >8 mmol·L^−1^ may be grounds for withholding resuscitation, as the highest recorded potassium level in an avalanche survivor was 6.4 mmol·L^−1^ (strong recommendation, moderate-quality evidence) [78]. Rescuers should also assess for risks posed by the environment to their own safety prior to commencing resuscitation efforts and opt for transfer of victims to ECLS or trauma centers, correspondingly.

Non-avalanche snow burial, which is composed of head-first immersion into tree wells or deep powder snow, is less represented within the published literature. Aside from case reports, only one review article and one small burial simulation study exist [79,80]. As such, data regarding self-extrication remain contradictory, specifically with respect to whether performance of rocking motions and removal of active gear (e.g., skis, snowboards) can truly complement survival efforts. No evidence exists on the use of transceivers, airbags, or AAPDs for better survival. Therefore, the WMS recommends avoiding outdoor activities near tree wells or deep snow as means of preventing non-avalanche snow burial, and in the event of an accident, a visual clue to rescuers can increase the chance of survival (weak recommendation, low-quality evidence).

The WMS has provided guidelines for prevention, field, and hospital management of avalanche- and non-avalanche-related snow burial, including rescuer guidelines that would optimize rescue efforts while ensuring safety. Nonetheless, as consequences of snow burials tend to be severe, the most important aspect of avalanche safety is their avoidance through appropriate education about routes and practices and employing good judgement.

## 2. Discussion

Since 2010, the WMS has accumulated evidence and presented guidelines regarding wilderness-environment-related injuries and illness. The guidelines address considerations from prevention to management and treatment of environmental illnesses. As new evidence has emerged, prevention and treatment strategies, as well as their respective gradings, have been updated for guidelines on acute altitude sickness, frostbite, and avalanche and non-avalanche snow burials, for which a summary can be found in Table 2.

**Table 2:** Summary of 2024 WMS altitude illness [2], frostbite [3], and avalanche/snow burial [4] guideline updates and appraisal of evidence.

Reported Guideline Recommendation	Reported Reason and Evidence	Grade	Study Cited	Study Type (Sample Size)	Reported Relevant Findings and Limitations
Altitude illness					
Inhaled budesonide should not be used for altitude illness prophylaxis.	Though small improvement in spirometry and oxygen saturation was reported, results could not be replicated in more methodologically sound clinical trials.	SRHE	Berger MM et al. (2017) [12]	RCT (*n* = 50); Intervention: 200 μg budesonide (*n* = 16), 800 μg (*n* = 17); Comparator: lactose-monohydrate (placebo; *n* = 17).	Relevant finding: Inhalation of budesonide does not have a significant effect on pulmonary gas exchange.
			Lipman GS et al. (2018) [13]	RCT (*n* = 103); Intervention: acetazolamide (*n* = 15), budesonide (*n* = 24); Comparator: placebo (*n* = 11).	Relevant finding: Budesonide was ineffective for the prevention of acute mountain sickness.
Acetaminophen should not be used for AMS prevention.	Study used improper control measures.	SRLE	Kanaan NC et al. (2017) [14]	Prospective randomized trial (*n* = 225 *); Intervention: acetaminophen (*n* = 113), ibuprofen (*n* = 112); Comparator: N/A excluded since enrolment.	Limitations: There was a lack of placebo-control group comparison. The participants were recruited at a high-altitude site, rendering any AMS incidence results potentially confounded by the preventive effect of preacclimatization.
Hypoxic tents are not recommended for facilitating acclimatization and preventing AMS.	On the basis of evidence from a placebo-controlled utility study that indicated a lower incident of AMS among users.	SRME	Dehnert C et al. (2014) [15]	RCT (*n* = 76); Intervention: hypoxic tent (*n* = 37; *n* = 21 for analysis); Comparator: normoxic tent (*n* = 38, *n* = 21 for analysis).	Relevant finding: Sleeping in a normobaric hypoxic tent for 14 consecutive nights reduced symptoms and incidence of AMS at higher altitudes later on. Limitations: Technical difficulties led to inconsistencies in hypoxic dose received by a number of participants.
Acetaminophen can be used to treat headache at high altitude but not to treat AMS or HACE.	A randomized controlled trial has found that acetaminophen can be used to alleviate high-altitude headache, but there is no evidence of its ability to effectively treat AMS or HACE.	SRLE	Harris NS et al. (2003) [16]	Prospective RCT (*n* = 74); Intervention: acetaminophen (*n* = 35); Comparator: ibuprofen (*n* = 39).	Relevant finding: Acetaminophen was as effective as ibuprofen in treating high-altitude headache, a common symptom of altitude illness.
Ibuprofen can be used to treat headache at high altitude but not to treat AMS or HACE.	A randomized controlled trial has found that ibuprofen can be used to alleviate high-altitude headache, but there is no evidence of its ability to effectively treat AMS or HACE.	SRLE	Harris NS et al. (2003) [16]	Prospective RCT (*n* = 74); Intervention: acetaminophen (*n* = 35); Comparator: ibuprofen (*n* = 39).	Relevant finding: Ibuprofen, which was chosen as the comparator in this study, was deemed effective in treating high-altitude headache, a common symptom of altitude illness.
No recommendation can be made regarding use of continuous positive airway pressure (CPAP) for AMS treatment.	Although feasibility of administering CPAP to treat AMS has been demonstrated, there is a lack of systemic research to support it.	N/A	Johnson PL et al. (2013) [17]	Two-part crossover study (*n* = 5; *n* = 14); Part 1: AMS prevention Intervention: overnight CPAP (*n* = 5); Comparator: no CPAP (*n* = 5); Part 2: AMS treatment Intervention: CPAP while awake + overnight CPAP (*n* = 14); Comparator: no CPAP at time of recruitment (*n* = 14).	Relevant finding: CPAP was useful for raising oxyhemoglobin saturation as part of treating AMS in high altitudes.
			Walmsley M. (2013) [18]	Case report (*n* = 1)	Relevant finding: The use of a portable CPAP device, in adjust to pharmaceutical treatments for AMS, HAPE and HACE, showed a marked improvement in oxygen saturation.
Salmeterol is not recommended for HAPE prevention.	Although in a randomized controlled trial it was demonstrated that salmeterol decreased HAPE incidence, it also caused side effects such as tremor and tachycardia. Furthermore, there is limited clinical experience to support such findings.	WRME	Sartori C et al. (2002) [19]	RCT (*n* = 37); Intervention: salmeterol (*n* = 18); Comparator: placebo (*n* = 19).	Relevant finding: Inhalation of salmeterol both decreased the incidence of pulmonary edema and alleviated hypoxemia-related symptoms of AMS. Three participants (two from the intervention group) reported tremor and/or nocturnal palpitations.
Nifedipine should be used for HAPE treatment when descent is impossible or delayed and reliable access to supplemental oxygen or portable hyperbaric therapy is unavailable.	One non-randomized unblinded study showed benefits of nifedipine in absence of other interventions, albeit at a dose that is no longer recommended. According to a prospective, cross-sectional study, addition of nifedipine to descent, oxygen, and rest did not provide additional benefit in terms of time to resolution of hypoxemia and radiographic opacities or hospital length of stay.	SRLE	Oelz O et al. (1989) [81]	Nonrandomized, unblinded study (*n* = 6); Intervention: nifedipine (*n* = 6); Comparator: N/A.	Relevant finding: Nifedipine (10 mg sublingually, 20 mg slow-release) was an effective treatment for all six participants with moderate-to-severe HAPE, resulting in better oxygenation, reduced alveolar arterial oxygen gradient and pulmonary artery pressure, as well as clearing of alveolar edema. No co-interventions were administered. As such, it may serve as an emergency intervention when descent is not possible.
			Deshwall R et al. (2012) [22]	Prospective cross-sectional study (*n* = 110); Intervention: oral nifedipine (alternating patients); Comparator: placebo (alternating patients).	Relevant finding: All HAPE patients fully recovered. Alongside the intervention and comparators, all patients were treated with descent, supplemental oxygen, and bed rest. For the monitored outcomes of oxygen saturation, duration of hospitalization, time needed for lung crepitation resolution, and radiographic findings, there were no statistically significant differences between the nifedipine and placebo groups.
CPAP may be considered for treatment of HAPE when supplemental oxygen or pulmonary vasodilators are not available or as adjunctive therapy in patients not responding to supplemental oxygen alone.	Although CPAP may be considered as an alternate or adjunctive avenue of therapy, it is not highly recommended due to a lack of systematic evidence and limitations on its overall utility and feasibility.	WRLE	Schoene RB et al. (1985) [82]	Experimental study (total: *n* = 17; HAPE group: *n* = 4; control group: *n* = 13); Intervention: CPAP (*n* = 17); Control: N/A.	Relevant finding: CPAP administered to HAPE subjects, at rest at high altitudes, resulted in improved gas exchange. In the control group, non-HAPE subjects participating in rigorous activity at high altitudes, CPAP resulted in greater arterial oxygen saturation at the expense of greater minute ventilation and heart rate. CPAP is recommended when descent is not a viable option.
Acetazolamide should not be used for treatment of HAPE.	Although there are clinical accounts of acetazolamide use, there are no systematic studies to support its use.	SRLE	Fagenholz PJ et al. (2009) [83]	Case series (*n* = 11); Intervention: bed rest, oxygen, nifedipine and acetazolamide (*n* = 11); Control: N/A.	Relevant finding: Multiple treatments were used on all patients, with some patients receiving additional pharmacological therapeutics. Methodology does not permit sound comparison of effectiveness.
			Jones BE et al. (2013) [84]	Retrospective case series (*n* = 56); Intervention: 83% received oxygen therapy, and 87% received nifedipine, 44% sildenafil, 32% dexamethasone, and 39% acetazolamide; Control: N/A.	Limitations: Methodology does not permit sound comparison of effectiveness. No comparison of inter-physician variability.
Although further studies are needed to determine the absolute efficacy of tPA for frostbite injury and to compare IV and intra-arterial tPA to IV prostacyclin, monitored administration of IV or intra-arterial tPA within 24 h of injury is recommended for the treatment of severe frostbite (Cauchy Grade 3 and 4), especially if iloprost is not available.	Through 2022, the recommendation for immediate intravenous or intra-arterial thrombolytic therapy for deep frostbite injuries had been further validated through evidence from one randomized controlled prospective trial (tPA plus iloprost, *n* = 16), one retrospective observational study (*n* = 125), three retrospective cohort studies (*n* = 59), eleven retrospective case series (*n*~200), and three case reports.	SRLE	Cauchy E et al. (2011) [39]	Randomized open-label trial (*n* = 47); Intervention: (1) aspirin and iloprost (*n* = 16) (2) aspirin, iloprost, and fibrinolytic agent, tPA (*n* = 16); Control: aspirin and buflomedil (*n* = 15).	Relevant finding: Risk of amputation following treatment of severe frostbite was significantly lower in the two intervention groups (0% and 19%, respectively) compared to the control group (60%). Study could not rule out additive effect posed by tPA in the second intervention outlined. The second intervention regimen should be considered on a case-by-case basis.
If available, appropriate imaging, including single photon emission computed tomography (CT)/CT, should be used to assess tissue viability and guide timing and extent of amputation.	Kraft et al. demonstrated the ability of single photon emission CT/CT to enable early and precise anatomic localization of nonviable tissue in a study assessing seven frostbite patients.	WRLE	Kraft C et al. (2017) [60]	Retrospective case series (*n* = 7, *n* = 19 frostbite injuries); Intervention: use of SPECT/CT to assess tissue viability in treatment planning for frostbite injuries; Control: N/A.	Relevant finding: In six patients, the intervention led to more distal amputation.
Consider iloprost for deep frostbite (Cauchy Grades 2–4) to or proximal to the distal interphalangeal joint within 72 h after rewarming but ideally as soon as possible.	A randomized trial assessing the efficacy of aspirin plus 1) buflomedil; 2) iloprost; or 3) intravenous tPA plus iloprost in 47 frostbite patients found a 0% amputation rate in the iloprost group. From randomized trials to case series, iloprost showed favourable effects if administered within 72 h of injury, with no serious side effects reported afterwards. Other bodies of evidence examining the use of iloprost for frostbite include a systematic review that included the aforementioned RCT (*n* = 1), one retrospective observational study (*n* = 90), and four case series.	SRLE	Cauchy E et al. (2011) [39]	Randomized open-label trial (*n* = 47); Intervention: (1) aspirin and iloprost (*n* = 16) (2) aspirin, iloprost, and fibrinolytic agent (*n* = 16); Control: aspirin and buflomedil (*n* = 15).	Relevant finding: Risk of amputation following treatment of severe frostbite was significantly lower in the two intervention groups (0% and 19%, respectively) compared to the control group (60%). Limitations include wide confidence intervals as well as a lack of ischemia documentation with appropriate diagnostic modalities prior to therapy initiation.
Avalanche and Non-avalanche Snow Burial Accidents					
An airbag that deflates after burial may help prevent asphyxia by creating an air pocket.	Maintenance of normal vital signs for a range of 49–60 min with airbag deployment prior to snow burial.	SRLE	McIntosh SE et al. (2020) [85]	Prospective cohort study (*n* = 12); Intervention: airbag deployment and subsequent snow burial with pre- and post-intervention monitoring of vital signs.	Relevant finding: 92% of participants were able to complete a 60 min snow burial with the use of air bags while maintaining oxygenation levels. Results reflect the potential of air bags to delay asphyxiation under proper condition only; if there is an oral or nasal obstruction, air bag deployment may not provide the same benefits.
Mountain rescue organizations should be prepared to manage multiple burial accidents.	As per AvaLife guidelines, CPR to be limited to 6 min in mass casualty incidents for victims who are normothermic and buried ≥150 cm deep.	EO	Genswein M et al. (2022) [86]	Experimental observational simulation field study (*n* = 1070 full burials).	Relevant finding: Use of Monte Carlo simulations of the AvaLife algorithm, derived from retrospective analysis of 1070 full burials and large prospective field tests, limiting CPR duration to 6 min may provide the “Greatest good for the greatest number” with a limited number of available rescuers. The main limitation is that this was an experimental study without human participants; the reported number of uses for this algorithm in real-life avalanche burials are low.
Core temperature (where measurable) or the Revised Swiss System can be used to estimate the risk of hypothermic cardiac arrest.	Level of consciousness has the best correlation with core temperature, which is a parameter the RSS uses to assess risk of cardiac arrest.	SRME	Musi ME et al. (2021) [87]	Evidence-based clinical guidelines on clinical staging of accidental hypothermia (Revised Swiss System, as part of the Recommendations of the International Commission for Mountain Emergency Medicine).	Limitations noted are as follows: (1) Overestimation of risk of cardiac arrest may occur with presence of conditions that may impair consciousness such as trauma, asphyxia, and HACE; (2) Use of level of consciousness as a single triaging factor poses a limitation when considering a single core temperature could manifest in different levels of consciousness.
EtCO_2_ < 10 mm Hg should not be used to predict a fatal outcome.	Hypothermic cardiac arrest is associated with a lower EtCO_2_ than normothermic cardiac arrest and should not be used as a measure to predict a fatal outcome.	SRHE	Darocha T et al. (2022) [88]	Case–control study of cardiac arrest patients (*n* = 131); Cohorts: hypothermic (*n* = 39) vs. non-hypothermic (*n* = 92).	Relevant finding: Hypothermic cardiac arrest is associated with decreased levels of ETCO_2_ and pH-stat PaCO_2_ than non-hypothermic cardiac arrests. Limitations include the following: (1) No adjustment for potential confounders such as quality of chest compressions and ventilation rate; (2) Possible systematic bias introduced by the potential time lag between the measurements of ETCO2 and PaCO2; (3) Only the first measurement of the parameters of interest were included in the analysis; (4) Statistical limitations with a study of 131 patients.
Provision of psychological assistance to avalanche victims and rescuers is recommended.	Avalanche rescuers are at risk of psychological burden and would benefit from formal support.	SRLE	Dolan N and Tedeschi C (2018) [89]	Qualitative study (*n* = 13 avalanche first responders included).	Relevant findings: (1) Among the 13 first responders interviewed, symptoms of substance use disorder, depression, anxiety, panic, acute stress disorder, post-traumatic stress disorder, and adverse effects on personal relationships were noted. (2) There is a lack of formal psychological support for avalanche first responders. Limitations include the following: (1) The study sample displayed underrepresentation of female and layperson responders and had geographical limitations; (2) Possibility of reporting bias, as interviews were conducted over the phone on sensitive topics that are difficult to communicate; (3) The variability in avalanche rescue experience confounded the participants’ experience of rescues and, thus, the psychological impact.
Avalanche accident registries should be used to improve the management of victims.	Quality indicators have been gathered by ICAR MedCom for management of avalanche victims.	SRME	Kottmann A et al. (2021) [90]	Consensus study Panel of 22 experts were consulted using a five-round modified nominal group technique to identify 23 quality indicators in avalanche rescue.	Limitations include the following: (1) Lack of reproducibility in consensus studies; (2) Bias of selection of experts (*n* = 16 ICAR Medcom members versus *n* = 6 non-members).

* value accounts for 107 participants. SRHE—strong recommendation, high-quality evidence; SRME—strong recommendation, moderate-quality evidence; SRLE—strong recommendation, low-quality evidence; WRHE—weak recommendation, high-quality evidence; WRLE—weak recommendation, low-quality evidence; WRME—weak recommendation, moderate-quality evidence; EO—expert opinion.

The WMS has also outlined areas of potential further investigation, which may lay the grounds for future updated guidelines. For acute altitude illness, as noted in the guidelines, the need for systematic research on adjunctive therapeutics and further research on illness management within the pediatric population has been outlined. As for frostbite injuries, advancement of medical inquiry into frostbite prevention medication, peri-thawing procedures for injury reduction, and post-thaw therapies for the improvement of long-term outcomes is encouraged. Similarly, it is difficult, and perhaps unethical, to conduct randomized trials for furthering research on avalanche injuries; therefore, better simulation technology would greatly propel the generation of sound evidence for prevention and treatment strategies regarding avalanche accidents. Further areas of investigation include assessment of the efficacy of implementing multiple preparedness measures, such as protective gear and air bag use, in combination as well as advances in search methods and emergency treatment procedures involved in advanced life support and ECLS.

Aside from the future areas of research outlined by the WMS, the guidelines may also benefit from further exploring the downstream clinical aspects of environment-related illness to reflect increasing clinical concerns of medical professionals, such as effects of climatologic events on health, extreme water-based devastation and its influence on food- and vector-borne diseases, as well as the clinical impact of air pollution and humidity on travelers [91]. Development of ever-more clinically -oriented guidance may require research efforts into more severe and uncommon cases being presented in medical settings. For example, in frostbite treatment, a clinical concern would be the phenomenon of afterdrop, or the decrease in core temperature as a result of rewarming peripheral blood vessels causing quicker mobilization of cold peripheral blood [81]. It would be worthwhile to explore whether there have been clinical accounts of this phenomenon that have contributed or will contribute to clinical guideline development. Bringing awareness to more clinical, case-based experiences and medical practices can not only provide precedence for treating rare cases but can also motivate further areas of research.

The limitations of the guidelines in their current form relate to some lack of clarity around specific questions—in PICO format—being addressed. As such, it is unclear if pediatric considerations have been fully explored and reviewed during the development process for both the avalanche and frostbite guidelines. Additionally, inclusion of a comprehensive search strategy with a literature cutoff date, even as a Supplementary File, would be informative and improve the accessibility of the guidelines. Involvement of additional stakeholders, including patients affected by the spectrum of cold- and altitude-related illness, the public, pediatric specialists, and allied health practitioners, particularly those working in the rehabilitation space, would be an asset to the subsequent iterations of each of the guidelines. A clear process and timeline for updating the guidelines could be considered, as could greater exposition around barriers and facilitators to uptake and implementation by stakeholder groups. The strengths of the guidelines include excellent clarity overall and unambiguous recommendations that are easily identified throughout each paper. The guidelines were universally developed with a high degree of rigor and used standardized approaches to evaluate the quality of the literature in order to assign the strengths of the recommendations accordingly. In each case, the guidelines are free of competing interests.

## 3. Conclusions

In 2024, the WMS provided updated guidelines on the prevention, treatment, and long-term management of acute altitude illness, frostbite injuries, and avalanche- and snow-burial-related injuries. These guidelines, which have been reviewed and graded based on the level of evidentiary support as well as their risk-to-benefit ratio, not only serve as systematically derived medical recommendations but also highlight the need for expansion of research efforts to fill gaps in the knowledge within the field of environmental medical science. Such concerted efforts can expand the scope of evidence-based practice in travel medicine and ideally standardize the implementation of recommendations within medical practices.

## Figures and Tables

**Table 1:** Summary of initial hospital management of frostbite injuries synthesized in the guidelines and respective guideline grades.

Reported Recommendation	Reported Guideline Grade
1. Treat hypothermia or serious trauma.	Strong recommendation, low-quality evidence
2. Rapidly rewarm in water heated and maintained between 37 and 39 °C (98.6 and 102.2° F) until area becomes soft and pliable to the touch (approximately 30 min).	Strong recommendation, moderate-quality evidence
3. Ibuprofen (12 mg·kg^−1^ per day divided twice daily).	Weak recommendation, low-quality evidence
4. Pain medication (e.g., opiate) as needed.	Strong recommendation, low-quality evidence
5. Tetanus prophylaxis.	Strong recommendation, low-quality evidence
6. Air dry (i.e., do not rub at any point).	Strong recommendation, moderate-quality evidence
7. Debridement: selectively drain (e.g., by needle aspiration) clear blisters and leave hemorrhagic blisters intact.	Weak recommendation, low-quality evidence
8. Topical aloe vera at each dressing change or every 6 h.	Weak recommendation, low-quality evidence
9. Dry, bulky dressings.	Strong recommendation, low-quality evidence
10. Elevate the affected body part if possible.	Strong recommendation, low-quality evidence
11. Systemic hydration.	Strong recommendation, low-quality evidence
12. Thrombolytic therapy: suggest IV or intra-arterial tPA for severe frostbite (Cauchy Grade 3 and 4) if less than 24 h after injury; use angiography for prethrombolytic intervention and monitoring of progress where available. Early, standardized tPA administration at remote, community hospitals prior to transfer to tertiary care centers may decrease rates of amputation.	Strong recommendation, low-quality evidence
13. Iloprost therapy: suggest for deep frostbite to or proximal to the distal interphalangeal joint (Cauchy Grades 2–4) up to 72 h after injury but ideally as soon as possible.	Strong recommendation, low-quality evidence
14. Clinical examination (plus angiography or technetium-99 bone scan if necessary) to assist determination of surgical margins. Evaluation by an experienced surgeon for possible intervention.	Strong recommendation, low-quality evidence

This table has been reproduced and slightly altered with permission from Elsevier (license No. 4854910032941).

## Data Availability

All data are presented in the manuscript.

## Supplementary Materials

The following supporting information can be downloaded at: https://www.mdpi.com/article/10.3390/ijerph22020284/s1, Table S1: Appraisal of updated WMS Clinical Practice Guidelines for environmental illnesses of travel (Acute Altitude Illness, Avalanche and Nonavalanche Snow Burial Accidents, and Frostbite) using the AGREE II framework.
