# Supplementary material for: What Is New in Altitude- and Cold-Related Illnesses of Travel: Appraisal and Summary of the Updated Guidelines from the Wilderness Medical Society
Source: Int J Environ Res Public Health. 2025 Feb 14;22(2):284. doi: 10.3390/ijerph22020284 (PMC11855094; doi:10.3390/ijerph22020284)
Supplement: Supplementary file 1 [file ijerph-22-00284-s001.zip › ijerph-3164983-supplementary.pdf]

**Supplemental Table 1.** Appraisal of updated WMS Clinical Practice Guidelines for environmental illnesses of travel (Acute Altitude Illness, Avalanche and Nonavalanche Snow Burial Accidents, and Frostbite) using the AGREE II framework.

| AGREE II Item | Mean Appraisal Scores |
|---|---|
| **Domain 1.** Scope and Practice | |
| 1. The overall objective(s) of the guideline is (are) specifically described. | 7.00 |
| 2. The health question(s) covered by the guideline is (are) specifically described. | 4.50 |
| 3. The population (patients, public, etc.) to whom the guideline is meant to apply is specifically described. | 5.50 |
| Aggregate Domain Scores: Domain 1 | 5.67 |
| Scaled Domain Scores: Domain 1 | 78% |
| **Domain 2.** Stakeholder Involvement | |
| 4. The guideline development group includes individuals from all the relevant professional groups. | 5.50 |
| 5. The views and preferences of the target population (patients, public, etc.) have been sought. | 3.00 |
| 6. The target users of the guideline are clearly defined. | 5.50 |
| Aggregate Domain Scores: Domain 2 | 4.67 |
| Scaled Domain Scores: Domain 2 | 61% |
| **Domain 3.** Rigor of Development. | |
| 7. Systematic methods were used to search for evidence. | 5.50 |
| 8. The criteria for selecting the evidence are clearly described. | 5.00 |
| 9. The strengths and limitations of the body of evidence are clearly described. | 6.50 |
| 10. The methods for formulating the recommendations are clearly described. | 7.00 |
| 11. The health benefits, side effects, and risks have been considered in formulating the recommendations. | 7.00 |
| 12. There is an explicit link between the recommendations and the supporting evidence. | 7.00 |
| 13. The guideline has been externally reviewed by experts prior to its publication. | 6.00 |
| 14, procedure for updating the guideline is provided. | 3.50 |
| Aggregate Domain Scores: Domain 3 | 5.94 |
| Scaled Domain Scores: Domain 3 | 82% |
| **Domain 4.** Clarity of Presentation | |
| 15. The recommendations are specific and unambiguous. | 7.00 |
| 16. The different options for management of the condition or health issue are clearly presented. | 7.00 |
| 17. Key recommendations are easily identifiable. | 7.00 |
| Aggregate Domain Scores: Domain 4 | 7.00 |
| Scaled Domain Scores: Domain 4 | 100% |
| **Domain 5.** Applicability | |
| 18. The guideline describes facilitators and barriers to its application. | 4.50 |
| 19. The guideline provides advice and/or tools on how the recommendations can be put into practice. | 6.00 |

| | |
|---|---|
| 20. The potential resource implications of applying the recommendations have been considered. | 6.50 |
| 21. The guideline presents monitoring and/ or auditing criteria. | 7.00 |
| Aggregate Domain Scores: Domain 5 | 6.00 |
| Scaled Domain Scores: Domain 5 | 83% |
| **Domain 6.** Editorial Independence | |
| 22. The views of the funding body have not influenced the content of the guideline. | 7.00 |
| 23. Competing interests of guideline development group members have been recorded and addressed. | 7.00 |
| Aggregate Domain Scores: Domain 6 | 7.00 |
| Scaled Domain Scores: Domain 6 | 100% |
| **Overall Assessment** | |
| 1. Rate the overall quality of this guideline. | 6.00 |
| Scaled Domain Scores: Overall Assessment | 83% |
| 2. I would recommend this guideline for use. | Yes (2)<br>Yes, with modifications (0)<br>No (0) |
